# Bulbar Onset Generalized Myasthenia Gravis in an Elderly Patient: A Diagnostic Challenge

**DOI:** 10.7759/cureus.105555

**Published:** 2026-03-20

**Authors:** Befkadu Abay, Moushumi Mehjeba, Anushka Bajgamage, Sanjay Suman

**Affiliations:** 1 Geriatrics, Medway NHS Foundation Trust, Gillingham, GBR; 2 Acute Medicine, General Internal Medicine, Medway NHS Foundation Trust, Gillingham, GBR

**Keywords:** bulbar mg, late onset mg, mg-activities of daily living index, mg in the elderly, ocular myasthenia gravis

## Abstract

Myasthenia gravis (MG) can present with variable and atypical symptoms, particularly in older adults, where isolated bulbar involvement may mimic stroke or motor neuron disease. We report a case of an elderly patient with late-onset, acetylcholine receptor (AChR) antibody-positive generalized myasthenia gravis who initially presented with ptosis, followed by progressive dysphagia and dysarthria, and subsequently developed head drop. Electromyography (EMG) confirmed a neuromuscular junction disorder, and serology demonstrated markedly elevated AChR antibodies. Early initiation of pyridostigmine and corticosteroids led to rapid clinical improvement, with the Myasthenia Gravis Activities of Daily Living (MG-ADL) score decreasing from 11/24 to 0/24 within three weeks. This case highlights the importance of considering MG in elderly patients presenting with isolated bulbar symptoms and demonstrates the diagnostic value of electrophysiology and antibody testing for timely treatment.

## Introduction

Myasthenia gravis (MG) is an autoimmune neuromuscular disorder characterized by muscle weakness and fatigability. In most cases, the pathogenesis of MG is mediated by autoantibodies directed against the nicotinic acetylcholine receptor (AChR) at the neuromuscular junction. Less commonly, autoantibodies may target muscle-specific kinase (MuSK) or low-density lipoprotein receptor-related protein 4 (LRP4). These autoantibodies impair cholinergic transmission at the neuromuscular junction through several mechanisms, including functional blockade, receptor downregulation, complement-mediated destruction, and disruption of receptor clustering within the postsynaptic membrane. Clinically, MG is characterized by fatigable weakness affecting ocular, bulbar, respiratory, or limb muscles. The clinical manifestations vary considerably among individuals depending on the antibody subtype and the presence of thymoma. Because of this variability in presentation, the diagnosis of MG can sometimes be challenging. Isolated bulbar symptoms, such as dysphagia and dysarthria, are relatively uncommon [1] and are more frequently observed in men with late-onset MG. Here, we report a rare case of late-onset myasthenia gravis in an 83-year-old man who presented with progressive bulbar symptoms.

## Case presentation

Our patient initially developed intermittent neck pain over a three-month period, which was subsequently followed by progressive dysphagia to solids, fatigable dysarthria, and later the development of head drop. His symptoms fluctuated, worsening later in the day. He also described intermittent ptosis, which required manual elevation of the eyelids, and a 3-4 kg unintentional weight loss.

His past medical history was positive for hypertension, hypothyroidism, hyperlipidemia, and diet-controlled type 2 diabetes mellitus. Medications included bendroflumethiazide, levothyroxine, and atorvastatin. He was fully independent and lived with his partner.

**Figure 1:**
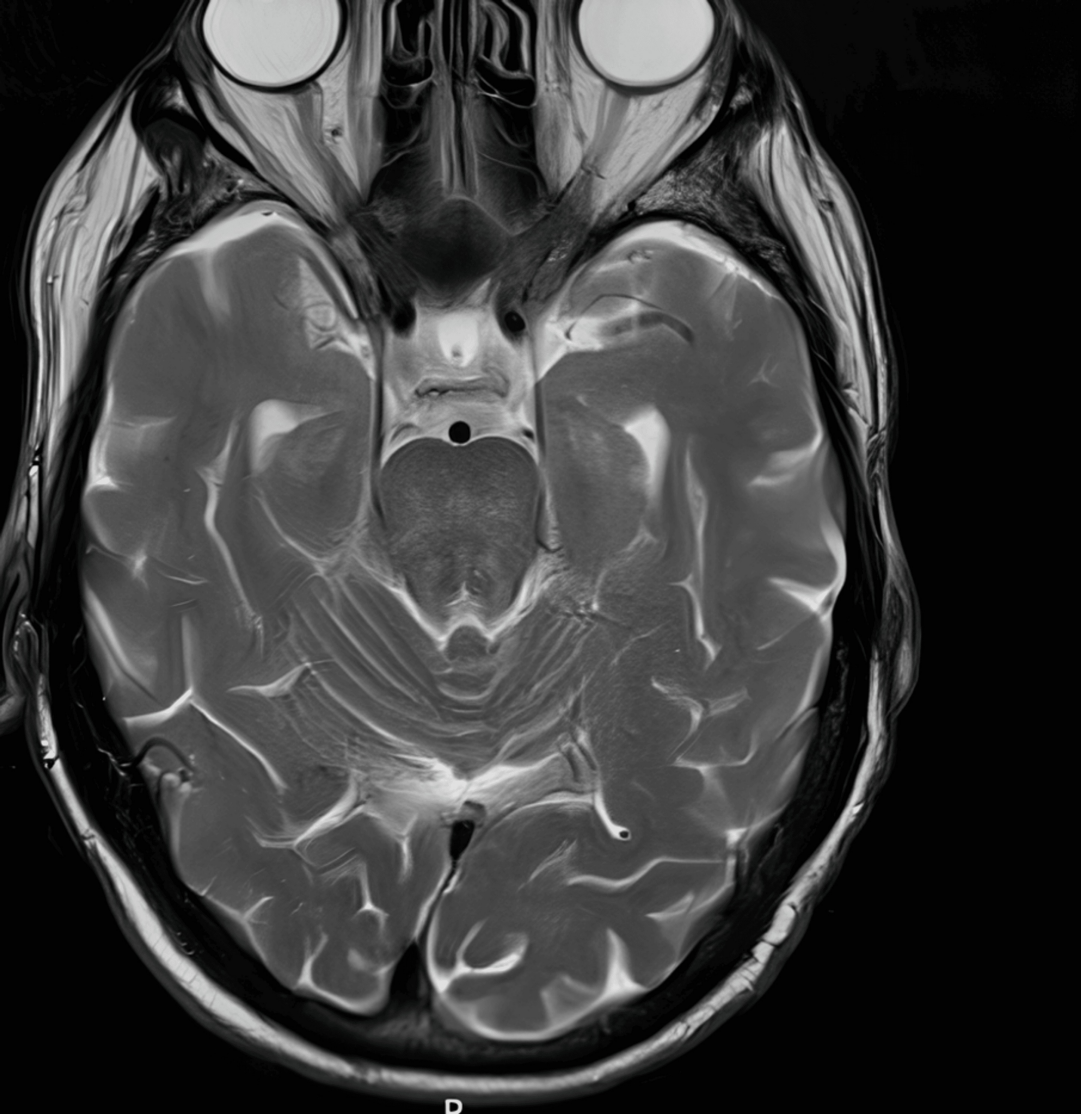
Brain MRI-axial section Axial T2-weighted MRI of the brain shows normal findings.

**Figure 2:**
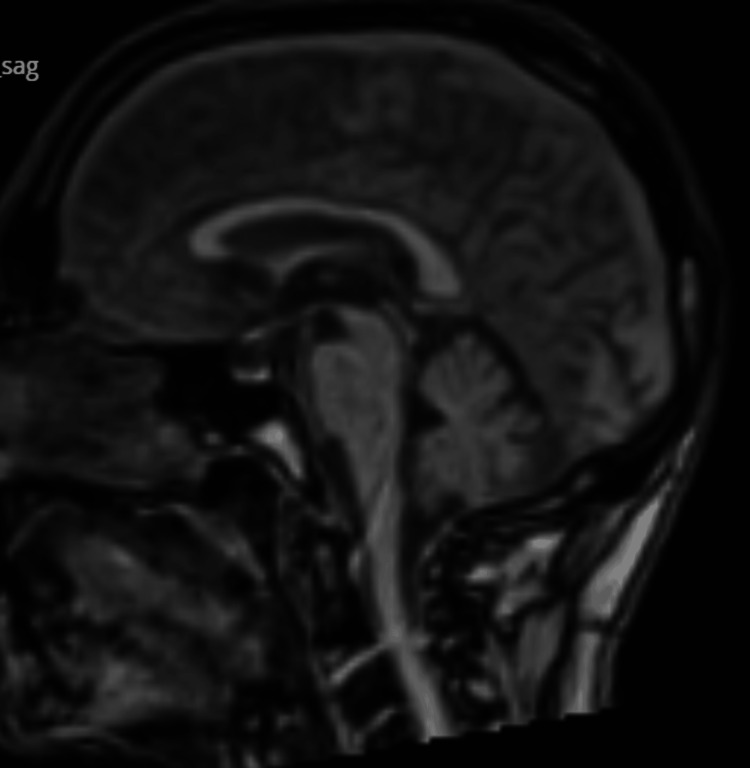
Brain MRI-sagittal section Sagittal T1‑weighted MRI of the brain demonstrates normal anatomy.

**Figure 3:**
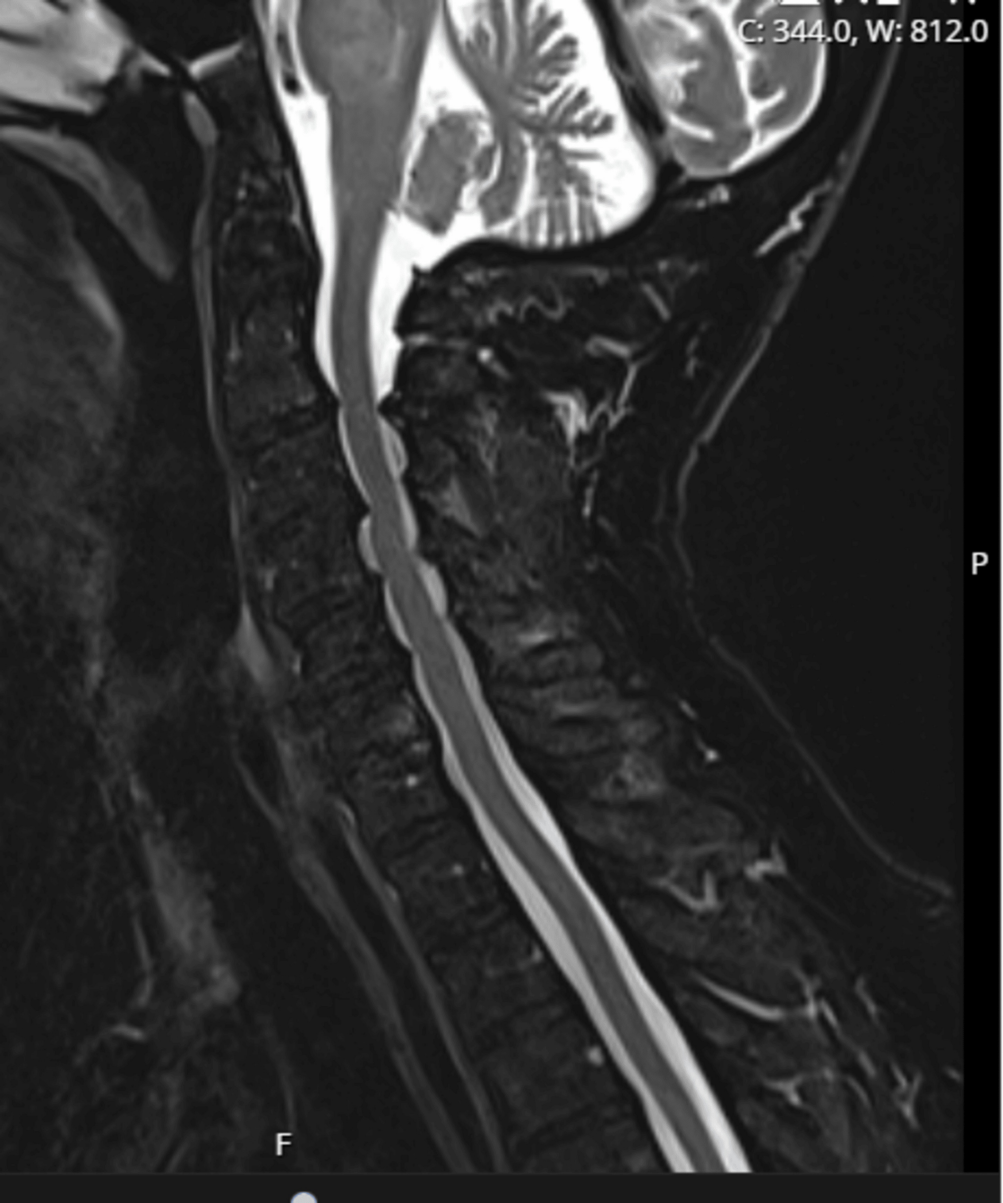
Sagittal T2 weighted MRI of the brainstem and cervical spine Sagittal T2-weighted MRI of the brainstem and cervical spine shows normal anatomy.

**Figure 4:**
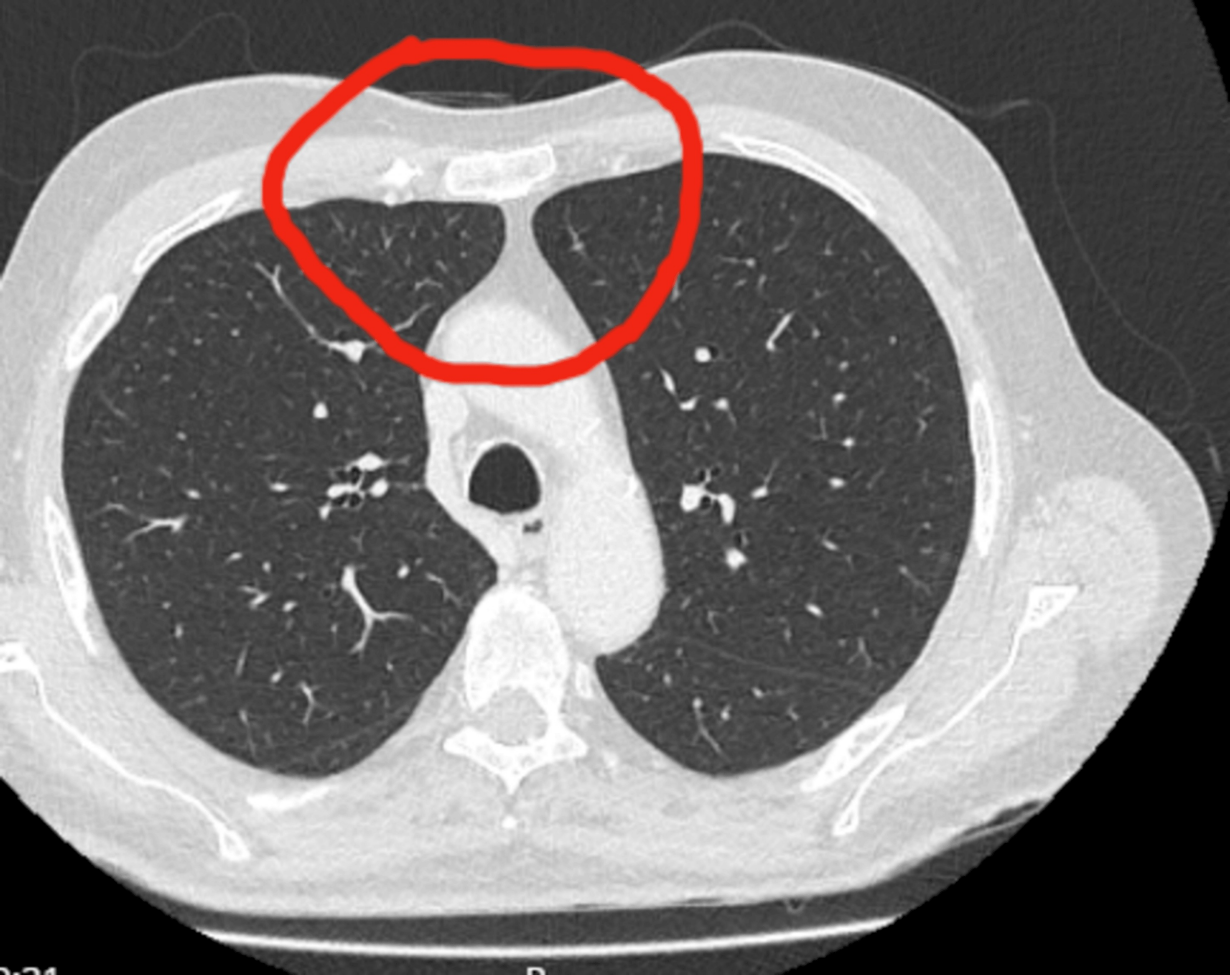
Axial CT scan of the chest An axial CT scan of the chest that does not show enlargement of the thymus gland (area marked in red).

On examination, vital signs were normal. He had bilateral facial weakness, bilateral fatigable ptosis, and dysarthric speech, which worsened on prolonged talking. Neck flexion was also weak. Both upper and lower limbs had normal tone, power, reflexes, coordination, and sensation. Single-breath count was 30. Given the absence of respiratory symptoms, formal forced vital capacity (FVC) measurement was not performed. The Myasthenia Gravis Activities of Daily Living (MG-ADL) score, assessed immediately after diagnosis to monitor treatment response, was 11/24. Investigation results are shown in Tables 1-5 and Figures 1-4.

**Table 1:** Blood test results The blood test showed a significantly high level of AChR antibody. AChR: acetylcholine receptor, Anti-CCP: anti–cyclic citrullinated peptide, FBC: full blood count, CRP: C-reactive protein, TSH: thyroid-stimulating hormone.

Blood test	Result	Reference value
AChR antibodies	267 x10^-10 ^mol	0-4 x10^-10 ^mol
Muscle tyrosine kinase	Negative	Negative
Anti-ganglioside antibody	Negative	Negative
Antinuclear antibody	Negative	Negative
Anti-CCP	1	1-7 u/mL
FBC	Within the normal limit	
CRP	1.6	0.0-5.0 mg/L
Kidney function test	Within the normal limit	
Electrolytes	Within the normal limit	
Liver function test	Within the normal limit	
TSH	3.71	0.3-4.8 MIU/L
Magnesium	0.73	0.7-1.00 mmol/L
Calcium	2.38	2.2-2.6 mmol/L
Phosphate	0.87	0.8-1.5 mmol/L
Folate	4.89	3-20 Ug/L
Vitamin B12	414	145-914 ng/L

**Table 2:** Repetitive nerve stimulation (RNS) result

Trial #	Label	Amp 1 (mV) O-P	Amp 5 (mV) O-P	Amp % Dif	Area 1 (mV·ms)	Area 5 (mV·ms)	Area % Dif	Rep Rate	Train Length	Pause time (min:sec)
Right abductor digiti minimi										
Tr 1	Baseline	7.92	8.18	3.3	26.23	25.20	-3.9	3.00	10	00:30
2	Post exercise							3.00	10	01:00
3	2 min Post							3.00	10	01:00
Right trapezius (upper)										
Tr 1	Baseline	3.99	3.11	-22.0	32.61	23.92	-26.7	3.00	10	00:30
Tr 2	Post exercise	3.51	3.04	-13.4	25.53	21.85	-14.4	3.00	10	01:00
Tr 3	2 min post	3.57	2.82	-21.0	26.54	20.45	-22.9	3.00	10	01:00

**Table 3:** Electromyography (EMG) report Electromyography (EMG): Left trapezius, deltoid, and extensor digitorum communis (EDC) show no fibrillation. Mild excess of polyphasic motor unit potentials (MUPs) and elevated jiggle.

Side	Muscle	Fibs/Psw	Fasc	Other	Amp	Dur	Poly	Recruitment	Interference Pat.
Left	Deltoid	None	None	None	N	N	N	N	Full
Left	Trapezius	None	None	None	N	N	N	N	Full
Left	EDC	None	None	None	N	N	N	N	Full

**Table 4:** Single fiber electromyography (SFEMG) study result

Muscle	Potential pairs	Abnormal pairs	Normal pairs	Jitter	Blocks
Extensor digitorum	5	60.0 %	40.0 %	105.0	60.0 %
Orbicularis oculi	7	0.0 %	100.0 %	137.3	0.0 %

Conclusion of electromyography and nerve conduction study results

Repetitive nerve stimulation demonstrated a significant decremental response in the right trapezius muscle, consistent with a postsynaptic neuromuscular junction disorder.

**Table 5:** Imaging results report (see Figures 1-4)

Imaging	Result
CT chest	Normal-No thymoma
MRI brain	Normal
MRI cervical spine	Normal

Differential diagnosis

The initial differential diagnoses included posterior circulation stroke, motor neuron disease (MND), oculopharyngeal muscular dystrophy, and myasthenia gravis. The diagnosis of myasthenia gravis was confirmed by the presence of fluctuating fatigability, positive electromyography findings, and elevated acetylcholine receptor antibodies. Posterior circulation stroke was ruled out due to a normal MRI and the presence of fluctuating symptoms. Motor neuron disease was excluded based on the absence of clinical signs of motor neuron involvement and the lack of characteristic EMG features. In addition, there was no known family history of neurological disorders.

Treatment

Following consultation with a specialist neurologist, the patient was commenced on pyridostigmine 30 mg three times daily, which was increased to 60 mg three times daily after one week. Prednisolone therapy was also initiated; at that time, the patient was taking 10 mg for the first three days of the week and 20 mg for the remaining four days. The patient responded well to the treatment.

**Figure 5:**
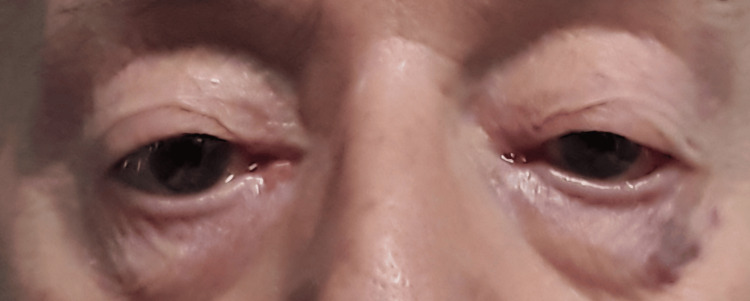
Picture of the eyes before treatment The picture shows significant bilateral ptosis.

**Figure 6:**
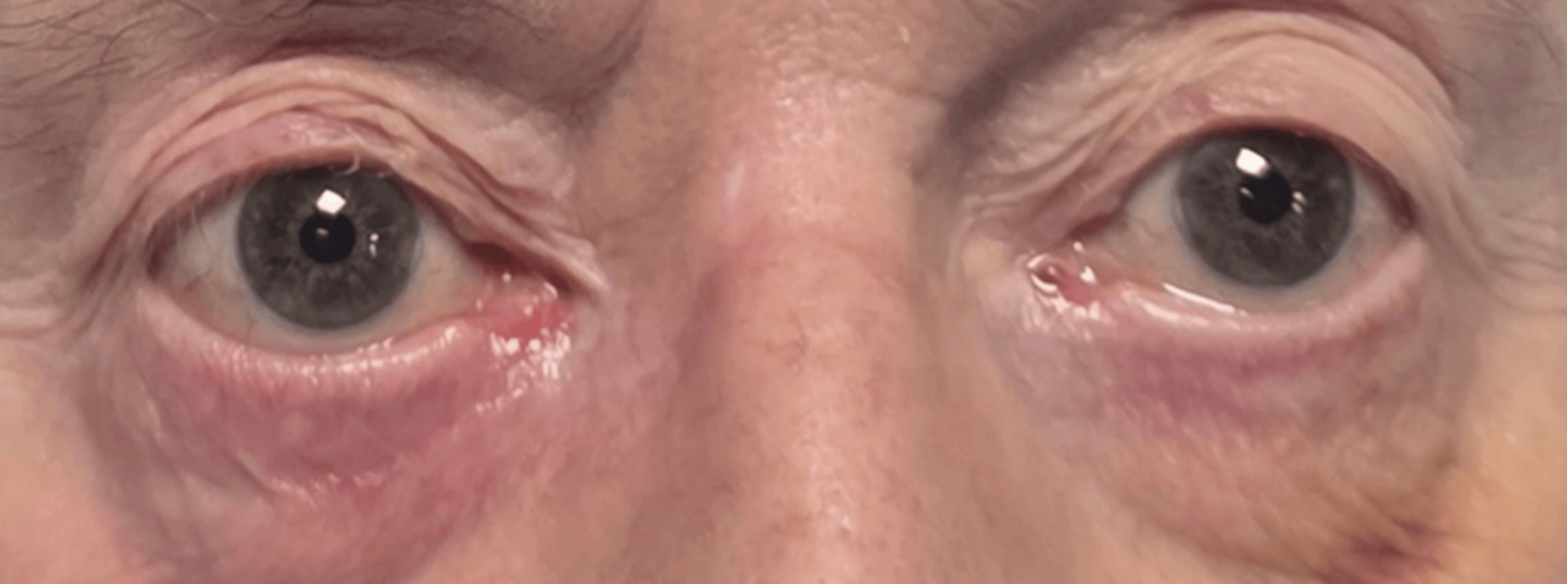
Picture of the eyes (taken seven days after the start of the treatment) The picture shows marked improvement of ptosis after the start of the treatment.

On the fifth day of treatment, there was a significant improvement in dysarthria, swallowing, and ptosis (see Figures 5, 6).

Outcome and follow-Up

Short-term follow-up was arranged after three weeks to assess the patient’s response to treatment, identify any side effects, and establish a long-term management plan. At review, the patient reported marked symptomatic improvement. Ptosis had resolved, and both dysarthria and swallowing had normalized. The patient also regained 2.5 kg in weight. The MG-ADL score improved to 0/24 (see Table 6 for MG-ADL scores over the three-week period). Mild intermittent head drop was slower to respond but has also shown improvement. Overall, the patient’s quality of life improved significantly, as evidenced by his ability to resume gardening, watch television comfortably, and eat solid food. The patient is currently under the care of the neurology and elderly care teams. He is taking pyridostigmine 60 mg four times daily and prednisolone 50 mg once daily, with appropriate gastric and bone protection. He continues to do well and remains asymptomatic.

**Table 6:** Myasthenia gravis activities of daily living (MG-ADL) index [2] comparison, before and after treatment (0=normal, 3=most severe, 1 and 2 are in between)

Activities of daily living	Before treatment	Day 5 of treatment	Day 21 of treatment
Talking	2	1	0
Chewing	2	2	0
Swallowing	2	2	0
Breathing	0	0	0
Impairment of the ability to brush teeth or comb hair	1	1	0
Impairment of the ability to arise from a chair	2	0	0
Double vision	0	0	0
Eyelid drop	2	1	0
Total score	11/24	7/24	0

## Discussion

Late-onset myasthenia gravis (LOMG) is increasingly recognised as an important subtype of myasthenia gravis, particularly in older adults. Epidemiological studies have demonstrated that the incidence of myasthenia gravis rises with age, with a peak between 60 and 80 years and a clear male predominance in late-onset disease [3,4,5]. Improvements in diagnostic awareness, serological testing, and an ageing population have contributed to the growing number of reported cases. We describe an 83-year-old patient who fits this demographic profile, presenting with acetylcholine receptor (AChR) antibody-positive generalized myasthenia gravis. This serological subtype is the most common form of LOMG, with AChR antibodies detected in approximately 80-90% of patients with generalized disease [6,7]. In contrast, muscle-specific kinase (MuSK) antibodies and other serological subtypes are considerably less frequent in the elderly population.

Clinical manifestations of LOMG can be variable and occasionally atypical, which may complicate early recognition. Ocular involvement remains the most common presenting feature, and several studies report that ocular symptoms occur more frequently in late-onset compared with early-onset disease [5,6]. Ptosis is the typical initial symptom and has been reported in up to 70-80% of patients at presentation [8]. However, a substantial proportion of patients with LOMG subsequently develop generalized disease with involvement of bulbar, facial, and proximal limb muscles. Bulbar manifestations, including dysphagia and dysarthria, have been reported in approximately 30% of cases and may sometimes represent the predominant presenting feature [9]. In our case, the patient presented with rapidly progressive bulbar symptoms over a period of weeks, highlighting the potential for atypical presentations in the elderly.

An additional unusual feature in this case was the presence of facial weakness without clear ophthalmoplegia. While facial muscle involvement is recognized in generalized myasthenia gravis, isolated facial weakness without significant extraocular muscle involvement is relatively uncommon and may contribute to diagnostic uncertainty [10]. Such atypical presentations can delay diagnosis, particularly when clinicians initially consider more common neurological conditions affecting older adults.

The diagnosis of myasthenia gravis in elderly patients can be particularly challenging because early symptoms are often subtle and may overlap with other neurological or systemic conditions common in this age group. Comorbidities such as cerebrovascular disease, Parkinsonism, and motor neuron disease may obscure the clinical picture, leading to diagnostic delay or misdiagnosis [8]. In addition, age-related physiological changes, polypharmacy, and frailty may further complicate clinical assessment. As a result, LOMG is thought to be under-recognized in older populations.

In this case, the patient’s progressive bulbar symptoms initially raised concerns for posterior circulation stroke and early bulbar motor neuron disease, both well-recognized mimics of myasthenia gravis. Posterior circulation stroke can present with acute dysphagia, dysarthria, and cranial nerve deficits, while bulbar-onset motor neuron disease may cause progressive dysarthria, dysphagia, and facial weakness. However, the fluctuating nature of symptoms, absence of upper and lower motor neuron signs, normal neuroimaging, and supportive electrophysiological and serological findings ultimately supported the diagnosis of myasthenia gravis. The presence of fatigable weakness, together with positive electromyography findings and elevated AChR antibody titers, further confirmed the diagnosis.

Early recognition of LOMG is clinically important because the condition is generally highly responsive to treatment. Symptomatic therapy with acetylcholinesterase inhibitors, such as pyridostigmine, often provides rapid improvement, while immunosuppressive therapy with corticosteroids or steroid-sparing agents may be required to achieve sustained disease control. Studies have shown that older patients with AChR antibody-positive myasthenia gravis generally respond well to standard treatment regimens, although careful monitoring is necessary due to the increased risk of medication-related adverse effects in the elderly population.

Overall, this case highlights the importance of considering myasthenia gravis in the differential diagnosis of progressive bulbar symptoms in older adults. Awareness of atypical presentations of LOMG, particularly when ocular signs are subtle or absent, may help prevent diagnostic delay. Prompt recognition and appropriate treatment can lead to significant symptomatic improvement and substantial gains in quality of life, even in very elderly patients.

Timely diagnosis requires both electrophysiology and serological testing. Repetitive nerve stimulation and single-fibre EMG confirmed a neuromuscular junction disorder, while markedly elevated acetylcholine receptor antibodies supported the diagnosis of MG [11,12]. Our case reinforces the importance of functional scales such as the MG-ADL, which is a very accessible, simple clinical tool to monitor treatment response [12,13].

Pyridostigmine is recommended as first-line pharmacologic therapy, with corticosteroids and additional immunosuppressants introduced when treatment goals are unmet [14]. Our patient’s clinical symptoms improved dramatically with pyridostigmine and corticosteroid therapy. Older patients often respond well to immunotherapy, with favorable outcomes reported in up to 60% of non-thymomatous LOMG patients receiving immunosuppressive treatment [15]. In our patient, early initiation of pyridostigmine and gradual steroid escalation led to functional improvement, reflected in the significant and rapid improvement in the MG-ADL score (see Table 6 for comparison of MG-ADL scores over three weeks).

Bulbar-onset myasthenia gravis in elderly patients warrants careful monitoring due to the risk of myasthenic crisis, which carries a reported mortality of 4.5-18.6% [16]. The diagnosis can be easily overlooked, as its presentation may mimic cerebrovascular events. Early recognition and prompt initiation of treatment, as demonstrated in this case, are essential to prevent respiratory failure and reduce the need for intensive care admission.

## Conclusions

This case report illustrates an atypical bulbar-predominant presentation of late-onset myasthenia gravis in an 83-year-old man, highlighting the diagnostic challenges of this condition in older adults. His initial bulbar and facial weakness closely mimicked stroke and motor neuron disease, both common in this age group. Fatigability and fluctuating symptoms, key diagnostic clues, were identified through careful history taking. A high index of clinical suspicion, combined with timely electrophysiological assessment and acetylcholine receptor antibody testing, facilitated early diagnosis and initiation of therapy, resulting in marked clinical improvement. This case underscores the importance of considering myasthenia gravis in elderly patients presenting with bulbar symptoms and emphasizes that early recognition and treatment are critical for preventing fatal complications such as myasthenic crisis and optimizing outcomes. The MG-ADL score proved to be a practical and reliable tool for monitoring treatment response in this patient.
